# Supplementary material for: Rice-Associated Rhizobacteria as a Source of Secondary Metabolites against Burkholderia glumae
Source: Molecules. 2020 May 31;25(11):2567. doi: 10.3390/molecules25112567 (PMC7321088; doi:10.3390/molecules25112567)
Supplement: Supplementary file 1 [file molecules-25-02567-s001.zip › Figure S2. Mass spectrum of 3-phenylpropanoic acid, TMS derivative.docx]

a

b

Figure S2: Mass spectrum of 3-phenylpropanoic acid, TMS derivative a) Experimental mass spectrum, b) Mass Spectrum Database NIST 1.7
