# Supplementary material for: Rice-Associated Rhizobacteria as a Source of Secondary Metabolites against Burkholderia glumae
Source: Molecules. 2020 May 31;25(11):2567. doi: 10.3390/molecules25112567 (PMC7321088; doi:10.3390/molecules25112567)
Supplement: Supplementary file 1 [file molecules-25-02567-s001.zip › Figure S4. NMR spectrum of the F4 fraction collected from the EtOAc extract of the BCB11 fer.docx]

Figure S4: NMR spectrum of the F4 fraction collected from the EtOAc extract of the biomass fermented with BCB11
