# Supplementary material for: Rice-Associated Rhizobacteria as a Source of Secondary Metabolites against Burkholderia glumae
Source: Molecules. 2020 May 31;25(11):2567. doi: 10.3390/molecules25112567 (PMC7321088; doi:10.3390/molecules25112567)
Supplement: Supplementary file 1 [file molecules-25-02567-s001.zip › Table S1. Consensus alignment of 16S rRNA gene sequence of BSB1 and BCB11 strains.docx]

Table S1. Consensus alignment of 16S rRNA gene sequence of BSB1 and BCB11 strains

Among the red markers the sequence of bases compared is shown. The only difference found is highlighted in yellow
