# Supplementary material for: Rice-Associated Rhizobacteria as a Source of Secondary Metabolites against Burkholderia glumae
Source: Molecules. 2020 May 31;25(11):2567. doi: 10.3390/molecules25112567 (PMC7321088; doi:10.3390/molecules25112567)
Supplement: Supplementary file 1 [file molecules-25-02567-s001.zip › Figure S6. Particle size distribution curves of emulsified EtOAc extracts.docx]

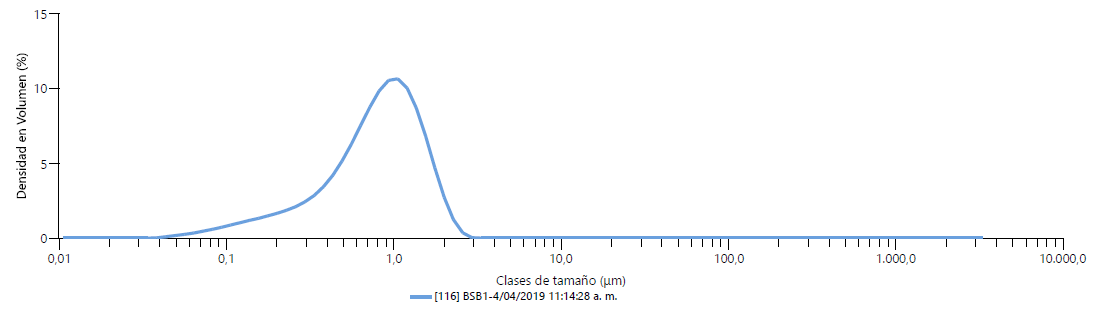

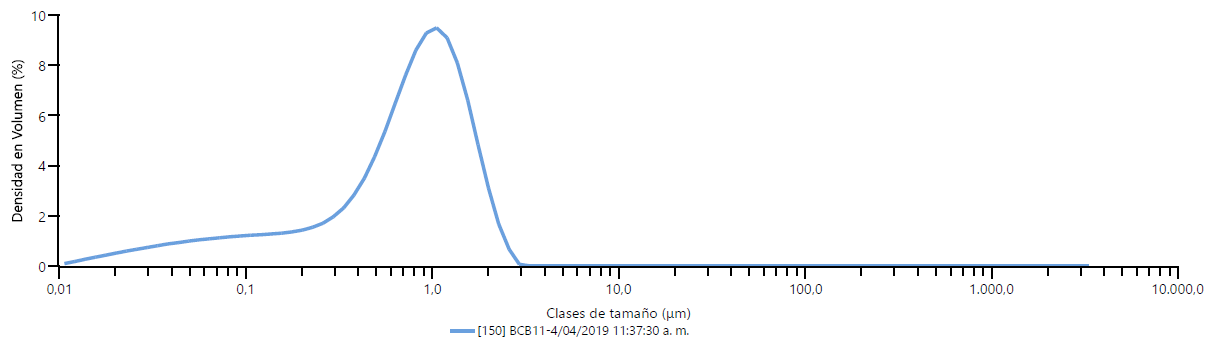

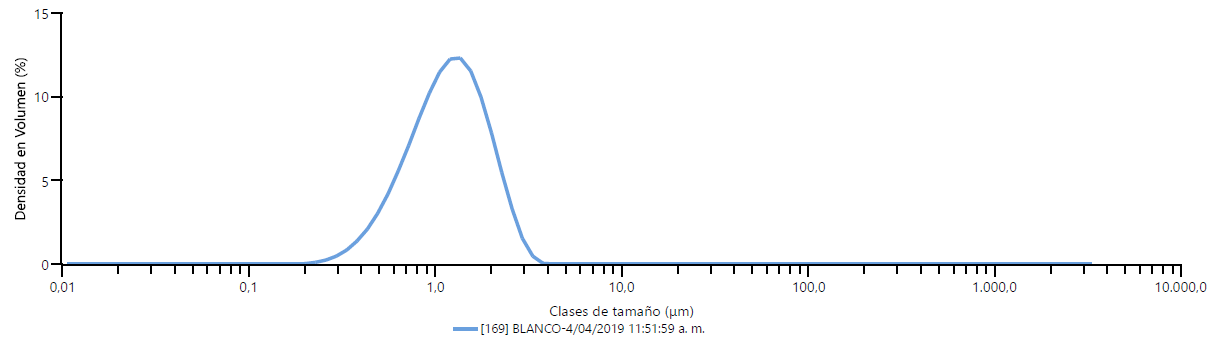


**a**

**b**

**c**

Figure S6: Particle size distribution curves of emulsified EtOAc extracts: **a**. Control, **b**. BSB1 and **c**. BCB11
