# Supplementary material for: Rice-Associated Rhizobacteria as a Source of Secondary Metabolites against Burkholderia glumae
Source: Molecules. 2020 May 31;25(11):2567. doi: 10.3390/molecules25112567 (PMC7321088; doi:10.3390/molecules25112567)
Supplement: Supplementary file 1 [file molecules-25-02567-s001.zip › Figure S5. Effects produced by Enterobacter strains on development of seedlings.docx]

**A**


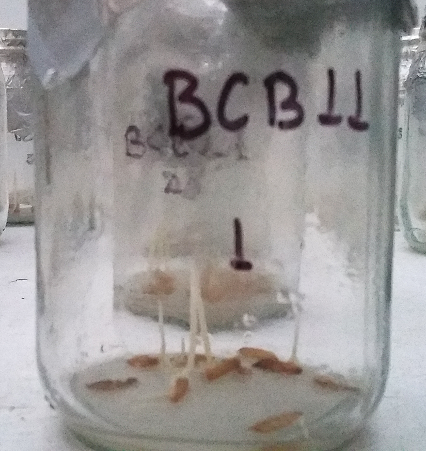

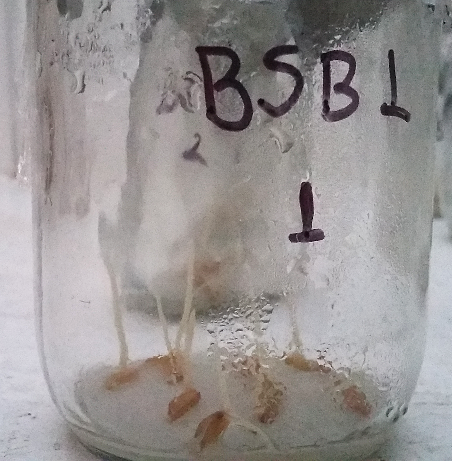

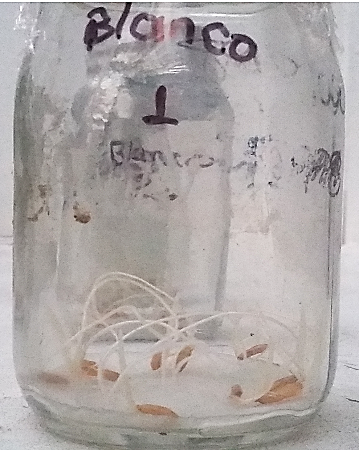


**B**


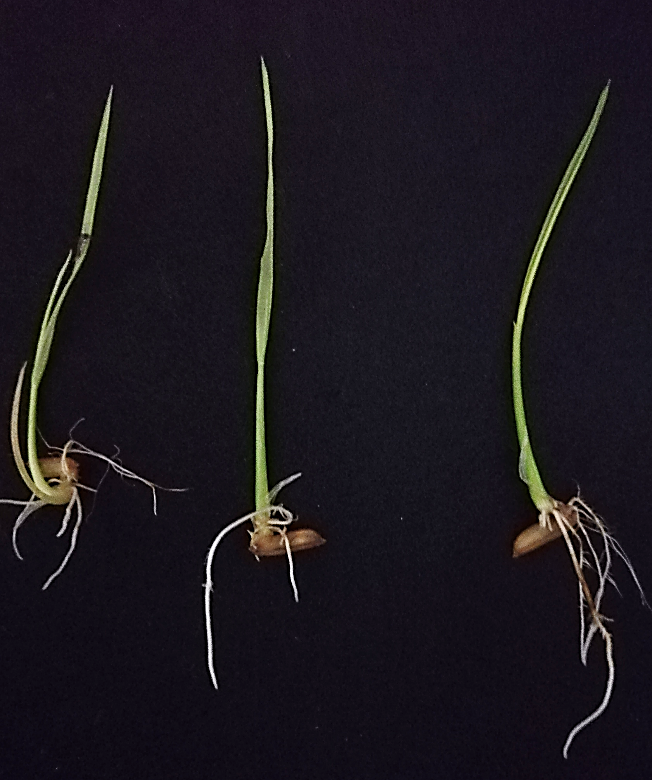


CONTROL

BCB11

BSB1

FEDEARROZ 2000


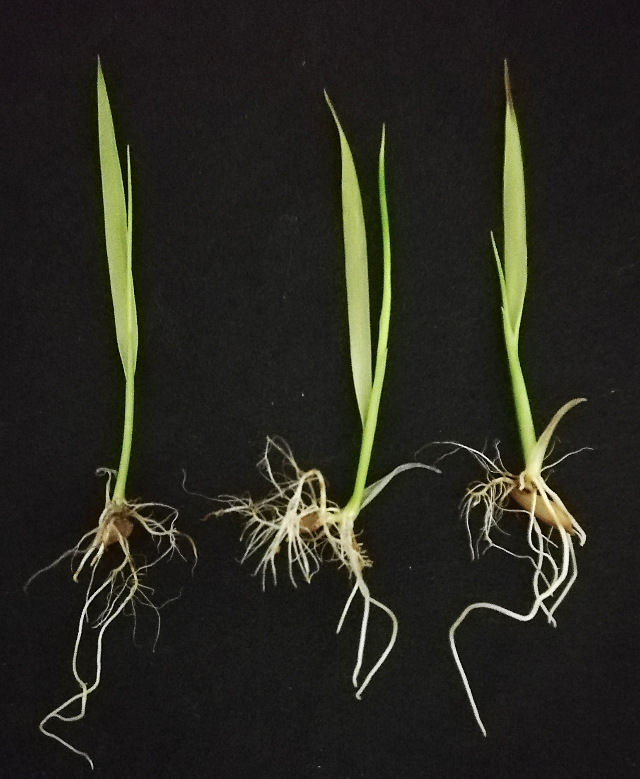


FEDEARROZ 67

CONTROL

BCB11

BSB1

Figure S5: Effects produced by Enterobacter strains on development of seedlings: A. Greenhouse flasks with germinated seeds; B. Seedlings with 15 days of development
