# Supplementary material for: Rice-Associated Rhizobacteria as a Source of Secondary Metabolites against Burkholderia glumae
Source: Molecules. 2020 May 31;25(11):2567. doi: 10.3390/molecules25112567 (PMC7321088; doi:10.3390/molecules25112567)
Supplement: Supplementary file 1 [file molecules-25-02567-s001.zip › Figure S1. Complete phylogenetic tree of BSB1 (MK715467) and BCB11 (MK715464) strains.docx]

Figure S1: Complete phylogenetic tree of BSB1 (*MK715467*) and BCB11 (*MK715464*) strains
