# Supplementary material for: Rice-Associated Rhizobacteria as a Source of Secondary Metabolites against Burkholderia glumae
Source: Molecules. 2020 May 31;25(11):2567. doi: 10.3390/molecules25112567 (PMC7321088; doi:10.3390/molecules25112567)
Supplement: Supplementary file 1 [file molecules-25-02567-s001.zip › Figure S7. Antagonistic activity of rhizobacteria BCB11 and BSB1 against B. glumae.docx]

| 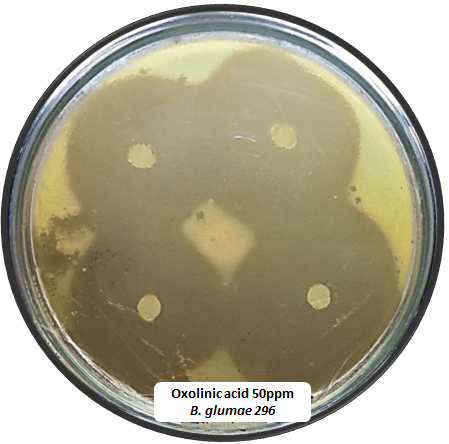  (**a**) | 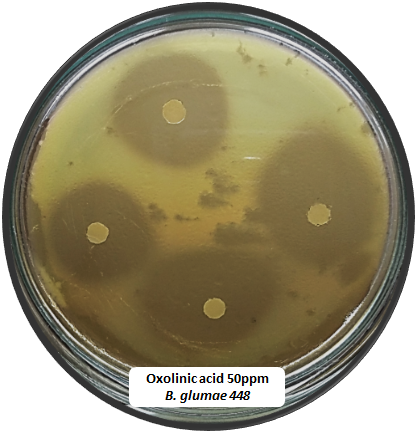  (**b**) | 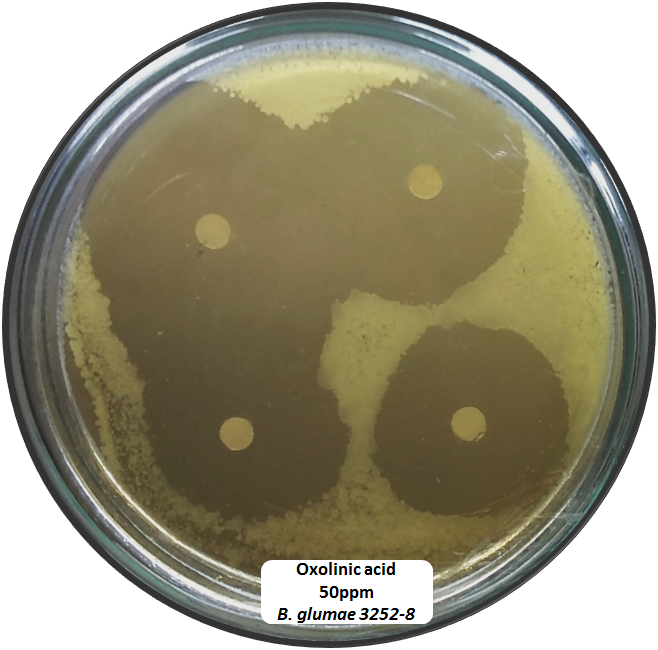  (**c**) |
| --- | --- | --- |

| 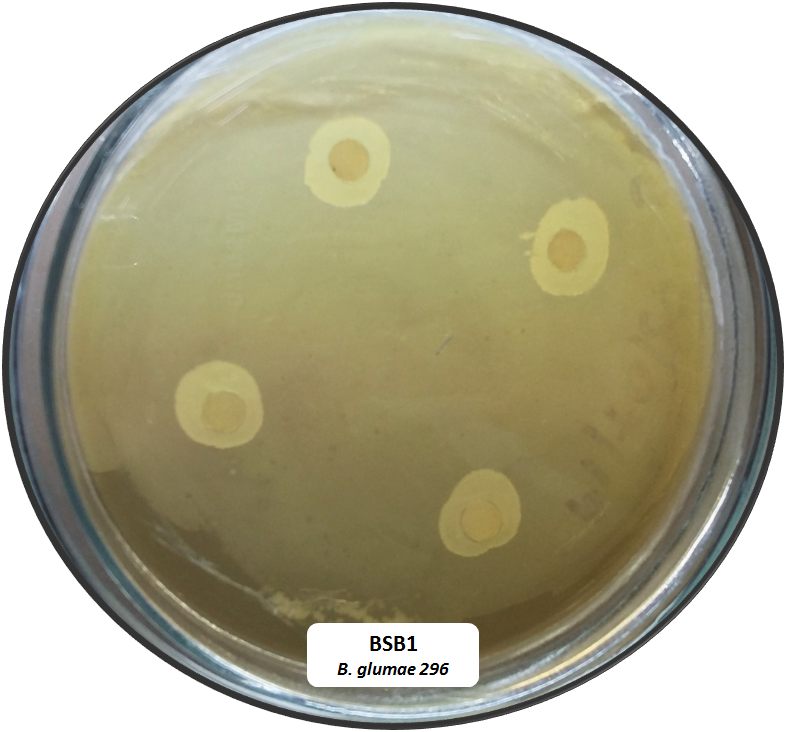  (**d**) | 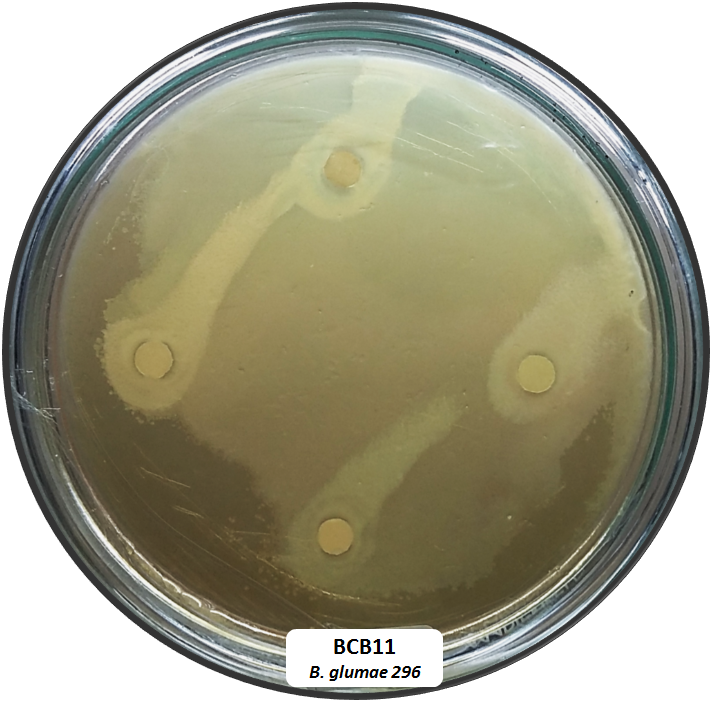  (**e**) | 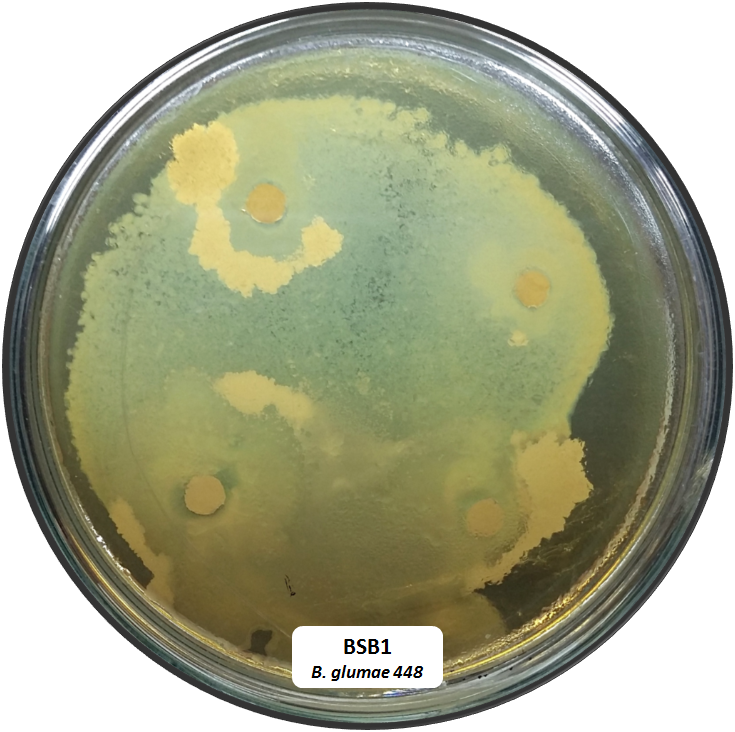  (**f**) |
| --- | --- | --- |

| 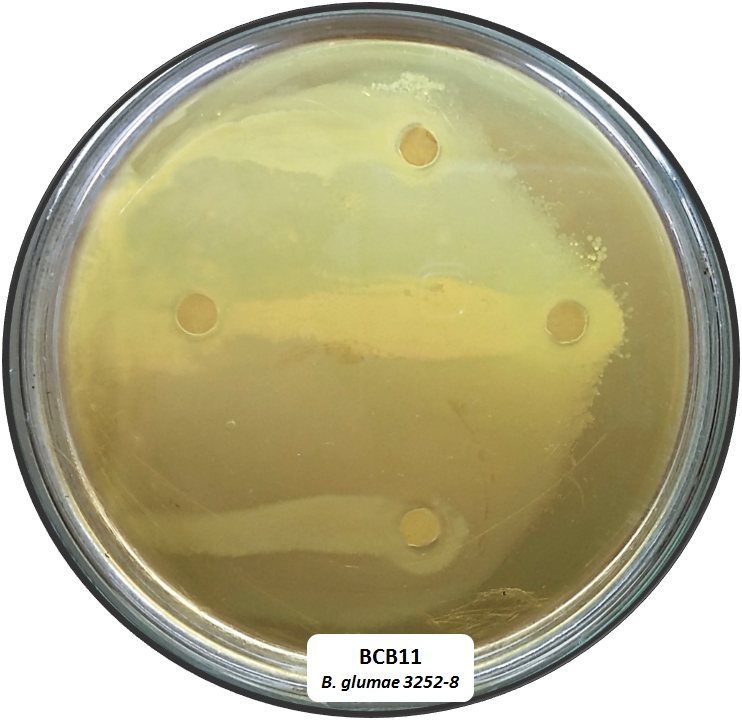  (**g**) | 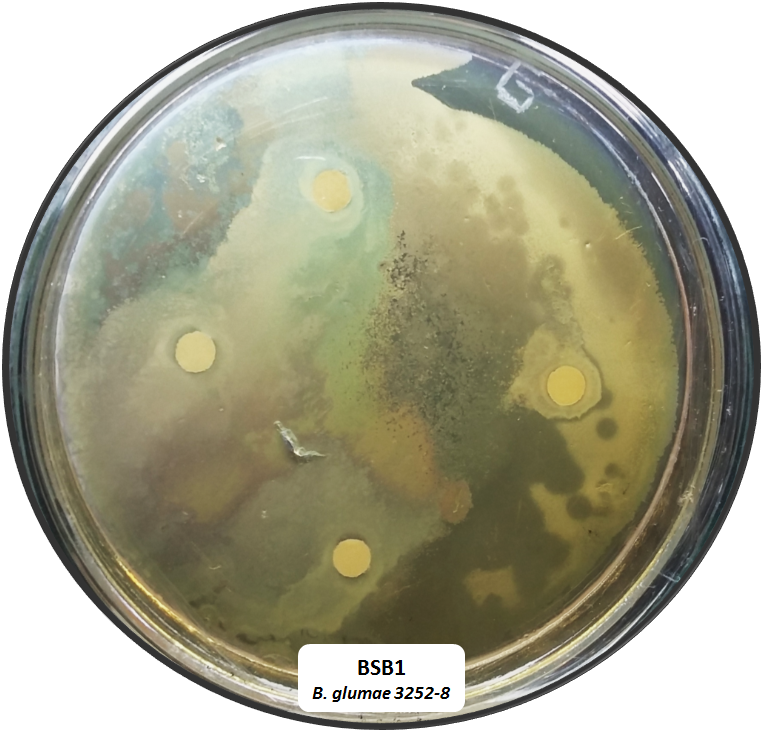  (**h**) |
| --- | --- |

Figure S7: Antagonistic activity of rhizobacteria BCB11 and BSB1 against *B. glumae*: **a**. Effect of oxolinic acid (50ppm) against *B. glumae* (296); **b**. Effect of oxolinic acid (50ppm) against *B. glumae* (448); **c**. Effect of oxolinic acid (50ppm) against *B. glumae* (3252-8); **d**. Effect of BSB1 against *B. glumae* (296); **e**. Effect of BCB11 against *B. glumae* (296); **f**. Effect of BSB1 against *B. glumae* (448); **g**. Effect of BCB11 against *B. glumae* (3252-8); **h**. Effect of BSB1 against *B. glumae* (3252-8)
