# Supplementary material for: Rice-Associated Rhizobacteria as a Source of Secondary Metabolites against Burkholderia glumae
Source: Molecules. 2020 May 31;25(11):2567. doi: 10.3390/molecules25112567 (PMC7321088; doi:10.3390/molecules25112567)
Supplement: Supplementary file 1 [file molecules-25-02567-s001.zip › Table S3. Identification of metabolites in the ethyl acetate extracts of the fermentation medium with BSB1.docx]

Table S3. Identification of metabolites by GC-MS in the ethyl acetate extracts of the fermentation medium of BSB1*.*

| **N°** | **Retention time (min)** | **Identification** | **Molecular formula** | **Molecular weight (g/mol)** | **Measured mass (*m/z*)** | **Relative area (%)** | **Characteristic ions (relative intensity, %)** |
| --- | --- | --- | --- | --- | --- | --- | --- |
| 1 | 3.408 | Lactic Acid, 2TMS derivative | C_9_H_22_O_3_Si_2_ | 234.11 | - | 0.03 | 219 (7.2); 190(16.3); 147(100); 117(81.2); 88(5.9); 73(94.7) |
| 2 | 5.498 | Benzoic acid, TMS derivative | C_10_H_26_O_2_Si_2_ | 234.15 | 234 | 0.04 | 193.95(9.6); 179(100); 135(41.1); 105(66.0); 77(57.9); 73(42.1) |
| 3 | 6.108 | Benzene acetic acid, TMS | C_11_H_16_O_2_Si | 208.09 | 208.1 | 3.00 | 208.1(1.3); 193(18.6); 164(23.8); 137(3.8); 91(15.3); 73(100) |
| 4 | 6.270 | Butanedioic acid, 2TMS derivative | C_10_H_22_O_4_Si_2_ | 262.11 | 262.10 | 0.04 | 262.1(0.8); 247(17.9); 147(100); 131(1.8); 73(48.8) |
| 5 | 6.396 | Catechol, 2TMS derivative | C_12_H_22_O_2_Si_2_ | 254.12 | 254.00 | 0.24 | 254(27.3); 239(8.3); 166(3.3); 151(4.8); 136(3.5); 73(100) |
| 6 | 6.643 | Uracil, 2TMS derivative | C_10_H_20_N_2_O_2_Si_2_ | 256.11 | 256.10 | 1.53 | 256.1(47.6); 215.1(100); 147(37.0); 126(6.4); 113(19.5) |
| 7 | 6.770 | 2,5-dihydroxy-3,6-dihydro-3,6-dimethylpyrazine | C_12_H_26_N_2_O_2_Si_2_ | 286.15 | 286.10 | 0.04 | 286.1(48.1); 271.1(100); 256(6.8) |
| 8 | 6.815 | Nonanoic acid, TMS derivative | C_12_H_26_O_2_Si | 230.17 | 230.10 | 0.04 | 230.1(1.6); 215.1(100); 145(12.3); 132(25.1); 117(70.9); 73(89.4) |
| 9 | 7.630 | 3-phenylpropanoic acid, TMS | C_12_H_18_O_2_Si | 222.11 | 222.1 | 28.58 | 222.1(27.3); 207(52); 104(100); 91(24.4); 77(10.1) |
| 10 | 7.742 | beta-Alanine, TMS | C_12_H_31_NO_2_Si_3_ | 305.17 | 305.3 | 0.13 | 305.2(1.7); 290.1(35.8); 248.1(92.3); 174(100); 147(41.9); 100(10.9); 86(16.1); 73(69.7) |
| 11 | 7.926 | Indole. TMS | C_11_H_15_NSi | 189.10 | 189.10 | 2.99 | 189.1(95.3); 174(100); 147(2.0); 117(7.7); 75(1.9); 73(37.9) |
| 12 | 8.017 | Decanoic acid, TMS | C_13_H_28_O_2_Si | 244.19 | 244.20 | 0.08 | 244.2(2.2); 229(100); 145(14.4); 129(30.1); 117(73.9); 73(81.6) |
| 13 | 8.650 | 4-Hydroxybenzyl alcohol, bis -TMS | C_13_H_24_O_2_Si_2_ | 268.13 | 268.10 | 0.14 | 268.1(50); 253(42.1); 223(4.9); 179(100); 149(7.8); 106(1.0); 89(3.4); 73(71.4) |
| 14 | 8.725 | N-(2-phenylethyl)-acetamide | C_10_H_13_NO | 163.14 | 163.14 | 0.90 | 163.1(24.7); 120(1.7); 104(100); 91(22.8); 72(16.2) |
| 15 | 9.449 | Tyrosol, 2 TMS | C_14_H_26_O_2_Si_2_ | 282.5 | 282.10 | 3.35 | 282.1(19.8); 267(13.3); 193(11.7); 179(100); 103(8.7);73(37.8) |
| 16 | 10.223 | 4-Hydroxybenzeneacetic acid, 2TMS | C_14_H_24_O_3_Si_2_ | 296.13 | 296.10 | 0.49 | 296.1(22.4); 281(20.5); 252(21.4); 179(35.3); 164(17.8); 149(5.3); 73(100) |
| 17 | 11.570 | Phloretic acid, 2 TMS | C_15_H_26_O_3_Si_2_ | 310.14 | 310.10 | 0.54 | 310.1(27.5); 295(8.5); 192(66.7); 179(100); 91(1.6); 73(36.3) |
| 18 | 11.665 | Benzyl benzoate | C_14_H_12_O_2_ | 212.08 | 212.00 | 0.06 | 212(31.2); 107(5.5); 105(100), 91(47.4); 77(32.3) |
| 19 | 12.408 | Myristic acid, TMS | C_17_H_36_O_2_Si | 300.25 | 300.20 | 0.13 | 300.2(0.3); 285.1(100); 243(1.1); 201(5.3); 145(24.4); 132(41.1); 117(83.4); 73(94.6) |
| 20 | 13.024 | Tryptophol, 2TMS | C_16_H_27_NOSi_2_ | 305.16 | 305.10 | 0.91 | 305(19.8); 290(4.3); 216(4.5); 202(100); 73(55.8) |
| 21 | 13.326 | 8-Phenyloctanoic acid | C_17_H_28_O_2_Si | 292.19 | 292.10 | 0.10 | 292.1(20.8); 277.1(100); 201(15.2); 145(24); 131(13.6); 117(76.4); 91(71); 73(53.2) |
| 22 | 13.711 | 3-Indolacetic acid, 2TMS | C_16_H_25_NO_2_Si_2_ | 319.14 | 319.10 | 1.29 | 319(24.4); 304(6.1); 202(100); 130(1.7);73(36.4) |
| 23 | 14.346 | Palmitic acid, TMS | C_19_H_40_O_2_Si | 328.28 | 328.20 | 0.49 | 328.2(7.2); 313(100); 201(5.5); 145(25.6); 132(38.6); 117(79); 73(74.3); 43(19.8) |
| 24 | 14.730 | (Z)-octadec-9-enenitrile | C_18_H_33_N | 263.26 | 263.20 | 0.06 | 263.2(14.5); 248(3.9); 139(2.5); 124(15.1); 97(38.7); 83(49.2); 69(71); 55(100); 41(90) |
| 25 | 15.228 | 5-Hydroxytriptophol, 3 TMS | C_19_H_35_NO_2_Si_3_ | 393.20 | 393.10 | 0.05 | 393.1(17.9); 378(1.3); 304(3.6); 290(100); 73(35.7) |
| 26 | 15.919 | Oleic Acid, TMS | C_21_H_42_O_2_Si | 354.30 | 354.20 | 0.07 | 354.2(9.9); 339.2(95.0); 222(16.0); 145(36.8); 132(16.4); 117(72.4); 73(100) |
| 27 | 16.129 | Stearic acid, TMS | C_21_H_44_O_2_Si | 356.31 | 356.30 | 0.69 | 356.3(7.7); 341(72.5); 215(58.2); 201(6.7); 145(28.2); 131(8.9); 117(68.3); 73(100) |
