# Supplementary figures and images for: Rice-Associated Rhizobacteria as a Source of Secondary Metabolites against Burkholderia glumae
Source: Molecules. 2020 May 31;25(11):2567. doi: 10.3390/molecules25112567 (PMC7321088; doi:10.3390/molecules25112567)

Figure S3: Spectrum FT-IR of F4 fraction from EtOAc extract from fermented biomass with BCB11

3015.7-3020 cm-1

650-850 cm-1


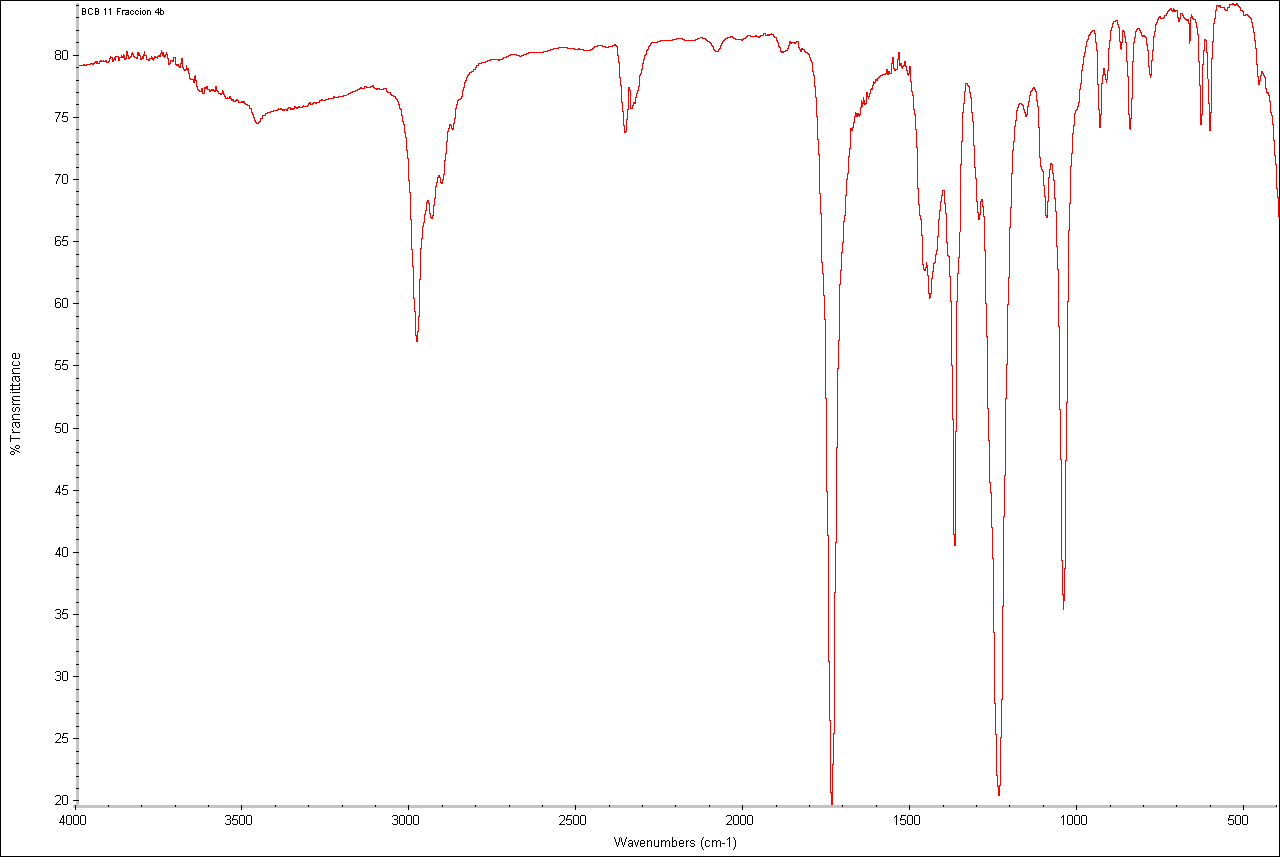

Supplement: Supplementary file 1 [file molecules-25-02567-s001.zip › Figure S3. Spectrum FT-IR of F4 fraction from EtOAc extract from fermented biomass with BCB11.docx]
